# The current state of African oncology research publication: how to increase Africa’s research impact

**DOI:** 10.3332/ecancer.2019.ed93

**Published:** 2019-07-23

**Authors:** Katie Foxall

**Affiliations:** ecancer, 13 King Square Avenue, Bristol, BS2 8HU, UK; a http://orcid.org/0000-0001-7511-4027

**Keywords:** Africa, oncology research, publishing, global inequalities, publication bias, open access

## Abstract

African oncology professionals face significant obstacles to getting their research published due to geographical bias, funding issues and lack of publication skills. There is a major inequity in the availability of high‐quality, local data from African countries and this makes it difficult for the continent’s governments to develop robust cancer-control policies based on the latest evidence. Both African and international journal publishers have a duty to work towards increasing the volume of African published research and making it freely available to all.

The global figure of new cancer cases is projected to rise to almost 22 million by 2030, with the burden in lower- and middle-income countries (LMICs) shifting from 59% to 65% of all cancer cases [1]—the greatest impact will clearly be in LMICs, many of which are ill-equipped to cope with this escalation in the number of people with cancer [2]. Africa will be hardest hit, and it is estimated that by 2030 cancer will kill over 1 million Africans each year [3]. Noncommunicable diseases have overtaken infectious diseases as the largest drain on productivity in Africa, accounting for 37% of the disease burden [4]. Radiotherapy is available in only 1/3 of African countries and oncology drugs are often unaffordable [5].

Populations and life expectancy, together with cancer incidence, are increasing in Africa. There is also an increased prevalence of risk factors associated with economic transition, including smoking, obesity, physical inactivity, and reproductive behaviours [6, 7]. Despite this growing cancer burden, cancer continues to be a relatively low public health priority in Africa, largely because of limited resources and other pressing public health problems, including communicable diseases such as AIDS/HIV infection, malaria, and tuberculosis [8]. 80% of global cancer deaths occur in LMICs but only 5% of total global spending on cancer care is expended in these regions. The problem is also in part due to a general lack of awareness among policy makers and the general public concerning the magnitude of the current and future cancer burden and its impact.

There is therefore an urgent need to educate the general public and patients regarding cancer prevention and treatment, and provide policy makers with high-quality, peer-reviewed, regionally appropriate evidence. This goes hand in hand with increasing the amount of research which is published and read by African oncology professionals so that they are both aware of the latest developments in global cancer research and are able to bring attention to the region-specific issues surrounding cancer prevention and care in Africa.

The World Cancer Declaration calls for the provision of innovative education and training for healthcare professionals (HCPs) to improve significantly, particularly in LMICs [9]. Africa has 25% of the global burden of disease but only 3% of the world’s healthcare workforce [10]. Many African oncology professionals need training in how to correctly assess and analyse the published results of research as well as education on how to develop skills in publishing their own research—this will translate to the development of robust cancer-control plans for each African country and improved patient outcomes.

Publication of research is an essential component of improving the skills of healthcare workers and addressing global inequalities in cancer communication, which inevitably leads to better patient outcomes. In 2017, five times as many research publications were published by the top ten countries in the world than by the bottom 200 [11]. Africa makes up around 15% of the global population, but only 0.7-1% of the world’s published research is by African authors [12]. There is a major inequity in the availability of high‐quality, local data from African countries [13]. Poor English language skills, lack of experience in applying the research method, and lack of funding are all part of the problem [14]. The inequality of global publication systems is also to blame, whereby research from the global North is seen as ‘mainstream’ or ‘international’ and research from the developing world is of ‘local’ interest only and therefore lacking sufficient impact for inclusion in the system unless it addresses issues that are of specific interest to Northern readers [15].

Editors of Northern journals, such as Richard Horton of *The Lancet*, are often constrained from publishing African authors, because this might reduce citations from the main readership—he has said: ‘The incentive for me is to cut off completely parts of the world that have the biggest health challenges … citations create a racist culture in journals’ decision-making and embody a system that is only about us (in the developed world)’ [16]. Of the ten top-cited articles ever published by *The New England Journal of Medicine*, three are trials of monoclonal antibody anti-cancer drugs that are largely unaffordable in Africa [17].

Research from Africa therefore has a very low international reach, and as a result, is not readily available to other researchers in the region or to the international scientific community. Being able to access the results of research is also a problem—the authors of a paper on the level of access that African emergency medicine researchers had to each other’s work found that ‘One sixth of research pertaining to Africa is being lost, only to be accessible to overseas researchers. Chances are it is likely to be research with recommendations that we need…and this is only due to cost obstacles’ [18].

The authors went on to say ‘A lot of people were saying, “We’d like to get into research, but one of the big stumbling blocks we have is, as soon as we start trying to do a literature search we hit these [paywalls]. Research underpins what we do in clinical medicine. If we have better access to research, especially our own research, we could provide better informed healthcare”’. Researchers from Africa and other LMICs are the least able to pay to access information or afford Article Processing Charges (APCs) so that they can publish their own research, resulting in ‘lost science’ in the form of information which is either not published or simply not made freely accessible to all. In some circumstances, access to the latest research findings can mean the difference between morbidity and mortality, and if regional research across Africa is rarely published, doctors will be unaware of specific data and best practices which would make a difference to outcomes for their patients.

One example of sharing best practices as part of information critical for regional development across Africa is advocating national palliative-care policies. Access to palliative care in the region remains extremely sparse despite the high disease burden—in the majority of African countries, most provision for palliation is from isolated centres of excellence without comprehensive integration into the different levels of the health system structure as recommended by the World Health Organisation (WHO) [19]. Published case studies listing the methods which work well in limited-resource settings from the centres which do exist, and guidelines on how best to form policy from the countries which do have national palliative-care policies, have the potential to make a significant impact on achieving palliative care for all in Africa [20]. The director of the African Palliative Care Association (APCA), Emmanuel Luyirika, has said ‘[publishing our research means] we are able to refer the donors, governments and academic institutions to the published best practices so that policy, planning and implementation are improved which results in benefits for patients. It has also enabled us to put our research and programme evidence across to the public.’ Luyirika went on to say that ‘It has improved our organisational profile as it is more credible with a body of publications’.

Careers of individual oncology professionals in Africa, as well as the profiles of organisations and universities, are also linked to the publication of research, with 94% of respondents to a survey of African healthcare professionals which* e*cancer carried out in early 2019 [21], saying that publishing in journals had benefitted their career. However, the majority of the 85 respondents (78%), had faced barriers to getting their research published, generally due to a lack of funding, inadequate research techniques, geographical bias and linguistic difficulties. A lack of available data which was relevant to their regional or racial context was also a problem. 85% had faced problems accessing the latest research and guidelines in order to be able to treat their patients—the biggest obstacle was cost, with 62% mentioning it.

Open access (OA) publishing goes some way to combating these problems as it provides free, immediate and permanent online access to the full text of an article, presenting researchers with easily accessible high-quality scientific resources essential to the rapid and efficient global communication of research findings [22]. Unfortunately, many open access journals charge an article publication charge which can cost up to thousands of dollars, thus creating another financial burden on poorly funded African researchers. There has also been a proliferation of ‘predatory journals’ which do not offer an acceptable level of peer review or publication quality and prey on authors from LMICs by charging lower APCs. These authors’ careers depend on publishing their research but they are often barred from publishing in high-level international journals. In South Africa, where authors are paid per published paper, many researchers are pushed into predatory publishing. Despite increased efforts to screen journals, many predatory publishers still make it onto accredited lists. The number of South African publications in predatory journals is now five times that of Brazil and the USA [23].

## What can be done to increase the amount of published African oncology research?

Organisations such as the International Network for the Availability of Scientific Publications (INASP) [24], produce online resources to support authors from LMICs through their AuthorAID initiative. They support the development and management of locally hosted online journal platforms in LMICs (including African Journals OnLine) and develop the capacity of editors and their institutions to promote the research published within their journals. They also run an online mentoring programme which pairs experienced mentors with researchers to support them at every stage of publication. The Hinari Access to Research for Health programme allows LMICs to gain access to a large collection of biomedical and health literature for free or at very low cost. However, individual international oncology journals also have a duty to support African authors to publish their research. Many journals offer fee waivers for authors from LMICs, but these often do not apply to all of the countries in Africa.

At *e*cancer, the journal team commissions articles which focus on areas where publication of the results of African research is most urgently needed, such as the implementation of vaccinations for liver and cervical cancers, tobacco-control policies for smoking-related cancers, and low-tech early detection methods for cervical cancer, as well as pain relief at the palliative stage. It is crucial that local experts are involved at every step of the publication process, so selected African oncology professionals are invited to join the Editorial Board and are also asked to serve as Guest Editors on special issues focusing on Africa. The journal team works closely with African authors during the pre-submission, peer review and publication processes, offering personalised support and helping them to develop their papers to meet international publishing standards. Only peer reviewers with a deep understanding of resource-limited settings are selected for these articles. African authors also receive free copyediting and open access publication with a CC BY copyright licence. *e*cancer, as a UK registered charity, continually fundraises to support these activities.

## Conclusion

The global inequities in the publication of research are never more starkly realised as they are in Africa, and this has a significant negative impact on the formation of evidence-based, robust cancer-control plans on the continent. With the increasing cancer burden in Africa, it is imperative that African oncology professionals develop publication skills and become fully involved in the global publication system, without having to face barriers to publishing their research such as geographical bias and lack of funding. By accessing this published region-specific information, cancer professionals in Africa are able to make informed decisions about the treatment of their patients. The international publishing community has a duty to support and collaborate with African researchers to make this a reality, with a consequent positive impact on population health and patient outcomes in Africa.

*‘If we stopped research entirely and never spent another dime on finding new cures, we could still save thousands of lives by doing one thing—breaking down barriers and sharing the knowledge that already exists’—*Dr Jill Biden, Co-Chair of the Biden Cancer Initiative.

## Conflicts of interest

The author is employed by *e*cancer, UK charity no. 1176307.

## Funding declaration

The author received no direct funding for this article.
